# International comparison of gender differences in Informant reports of cognitive decline in older adults

**DOI:** 10.1002/alz.092763

**Published:** 2025-01-09

**Authors:** Phillip A Cantu

**Affiliations:** ^1^ UTMB, Galveston, TX USA

## Abstract

**Background:**

The goal of this study is to examine how gender of research participants affects informants assessments of cognitive functioning in older adults. Informant assessments are important tools when clinicians diagnose cognitive decline and dementia and have been used to characterize highly impaired adults in population studies. Contextual and social factors for men and women in different countries may make informants more or less likely to report changes in functioning and cognitive declines at different levels of cognitive impairment.

**Method:**

his study examines gender differences in informant reports of cognitive function in different national contexts using data derived from the Harmonized Cognitive Assessment Protocol (HCAP) of the Health and Retirement Study (HRS)(n = 2,966) and the Mexican Cognitive Aging Study (MexCOG)(n = 1,750). Participants in the HCAP in each nation undergo a direct assessment of cognitive function as well as an informant assessment of cognitive functioning. The HCAP and MexCog contain 19 comparable informant questions and we control for age, gender, education, directly assessed cognition using the Mini Mental Status Examination (MMSE), informant type (spouse, child, or other) and coresidence status.

**Result:**

Both nations have gender differences in informant reports of memory impairments, but now always in the same direction. Informants were more likely to report conversation impairments for women in Mexico. There are fewer gender differences in orientation questions for each nation, may be related to high level of impairment. There were more gender differences in reports of functional limitation in the U.S. than in Mexico. Additionally we found that the gender differences were specific to each nation.

**Conclusion:**

Informants were MORE likely to report symptoms of cognitive decline in the U.S. than in the Mexico. Informants were more likely to report memory issues (forgets were things are kept, forgets where they put things), and difficulty with chores for women in both countries. Gender differences were not the same in each nation. Relative to Mexico, informants in the U.S. were less likely to report memory problems and talking about the past for women and more likely to report forgetting when last saw informant and difficulty handling money.

